# Systemic treatment in patients with Child–Pugh B liver dysfunction and advanced hepatocellular carcinoma

**DOI:** 10.1002/cam4.6033

**Published:** 2023-05-10

**Authors:** Frederico Costa, Bertram Wiedenmann, Christoph Roderburg, Raphael Mohr, Ghassan K. Abou‐Alfa

**Affiliations:** ^1^ Centro de Oncologia Hospital Sírio‐Libanês São Paulo Brazil; ^2^ Department of Hepatology and Gastroenterology Charité University Hospital Berlin Germany; ^3^ Clinic for Gastroenterology, Hepatology and Infectious Diseases University Hospital Düsseldorf Düsseldorf Germany; ^4^ Memorial Sloan Kettering Cancer Center New York New York USA; ^5^ Weill Medical School at Cornell University New York New York USA

**Keywords:** Child–Pugh B, cirrhosis, hepatocellular carcinoma, immuno‐oncology, multi‐kinase inhibitors

## Abstract

Hepatocellular carcinoma (HCC) is a major cause of death among patients with liver cirrhosis. The rise of immuno‐oncology has revolutionized treatment for advanced HCC. However, most pivotal randomized controlled trials have excluded patients with moderate liver dysfunction (Child–Pugh–Turcotte B), despite the high incidence of liver disease in patients with HCC at the time of diagnosis. Overall survival in patients with HCC and moderate liver dysfunction treated with sorafenib has been found to be only approximately 3–5 months, underlining the need for improved treatment algorithms for this increasingly important subgroup of patients. In this review, we summarize available data on the treatment of patients with HCC and moderate liver dysfunction. Opportunities, as well as clinical challenges, are discussed in detail, highlighting potential changes to the therapeutic landscape.

## 1 INTRODUCTION

Chronic liver disease is a direct cause of around 2 million deaths worldwide every year^1^ and is associated with viral infections including hepatitis B and C, nonalcoholic liver disease (mainly secondary to diabetes and morbid obesity), alcohol consumption, and other metabolic and exposure factors.^2^, ^3^, ^4^, ^5^, ^6^ The severity and prognosis of liver disease is usually assessed using the Child–Pugh–Turcotte (also known as the Child–Pugh [CP]) classification system.^7^, ^8^ Hepatocellular carcinoma (HCC) is the fourth most common cause of death among patients with cirrhosis,^9^, ^10^, ^11^ which coexists with HCC in most cases. The risk of HCC development at 15 years after the onset of cirrhosis is greater than 60%, depending on the underlying liver disease.^12^, ^13^

The balance of HCC and cirrhosis determines proportional prognostic relevance, and their separate clinical courses should be weighed for personalized patient management.^14^ Despite screening programs for high‐risk patients, 50% of new cases of HCC occur in patients diagnosed with advanced stages of the disease.^15^ Patients with severe liver damage (CP C) should be considered for palliative care, but are usually ineligible for clinical trials. For patients with HCC and well‐preserved liver function (CP A), transarterial chemoembolization alone or in combination with systemic therapies is an effective treatment option. Patients with advanced HCC and well‐preserved liver function (CP A) have seen considerable improvement in treatment options since 2018, following completion of several Phase 3 studies in this population, including the studies IMbrave150, HIMALAYA, CheckMate 459, COSMIC‐312, REFLECT, and LEAP‐002.^16^, ^17^, ^18^, ^19^, ^20^, ^21^ There are now numerous therapeutic options in first, second, and later lines of therapy^22^ and the introduction of effective immunotherapies has had a significant impact on patient outcomes.^15^, ^23^

Several groups of patients with intermediate and advanced HCC are, however, often excluded from clinical trials.^24^ Active viral infection, active esophageal varices, moderate liver dysfunction (CP B), and limiting renal function are common exclusion criteria in most of the pivotal clinical trials. These patients usually present with poorly compensated liver disease and/or poor performance status (Eastern Cooperative Oncology Group performance status [ECOG PS] of 2) and empirical evidence of efficacy and safety is sparse in this population. Despite this, most of the approved drugs used to treat advanced HCC have been offered in this population. Here, we describe and discuss the current data on systemic treatment in patients with HCC and moderate liver dysfunction.

### 1.1 Classification of liver function

Historically, the CP system has been the most widely used tool for classifying liver function. The CP classification is based on clinical signs of liver function, including ascites, encephalopathy, bilirubin, albumin, and prothrombin time/international normalized ratio. Each measure is scored 1–3 according to severity, and patients are classified as CP A (5–6 points) for normal liver function, CP B (7–9 points) for moderate dysfunction, or CP C (10–15 points) for severe dysfunction.^25^ Subclassifications of CP have been proposed, but need further validation.^26^ The CP system is frequently used as the basis for inclusion criteria in clinical trials in intermediate and advanced HCC.^23^, ^27^

More recently, the albumin–bilirubin (ALBI) system for classification of liver function has been introduced as an alternative method for predicting treatment efficacy and survival in patients with liver damage and HCC.^8^, ^25^, ^27^ The ALBI system assigns patients to one of three prognostic categories (ALBI 1–3) according to:

$$\begin{array}{cc} \text{Linear predictor}=\; & \left(\log10\;\text{bilirubin}\;\left[\mathrm{mol}/L\right]\times 0.66\right)\\ & +\left(\text{albumin}\;\left[g/L\right]\times 0.085\right) \end{array}$$

where Grade 1 ≤−2.60; Grade 2 >−2.60 and ≤−1.39; and Grade 3 >1.39.^25^

**FIGURE 1:**
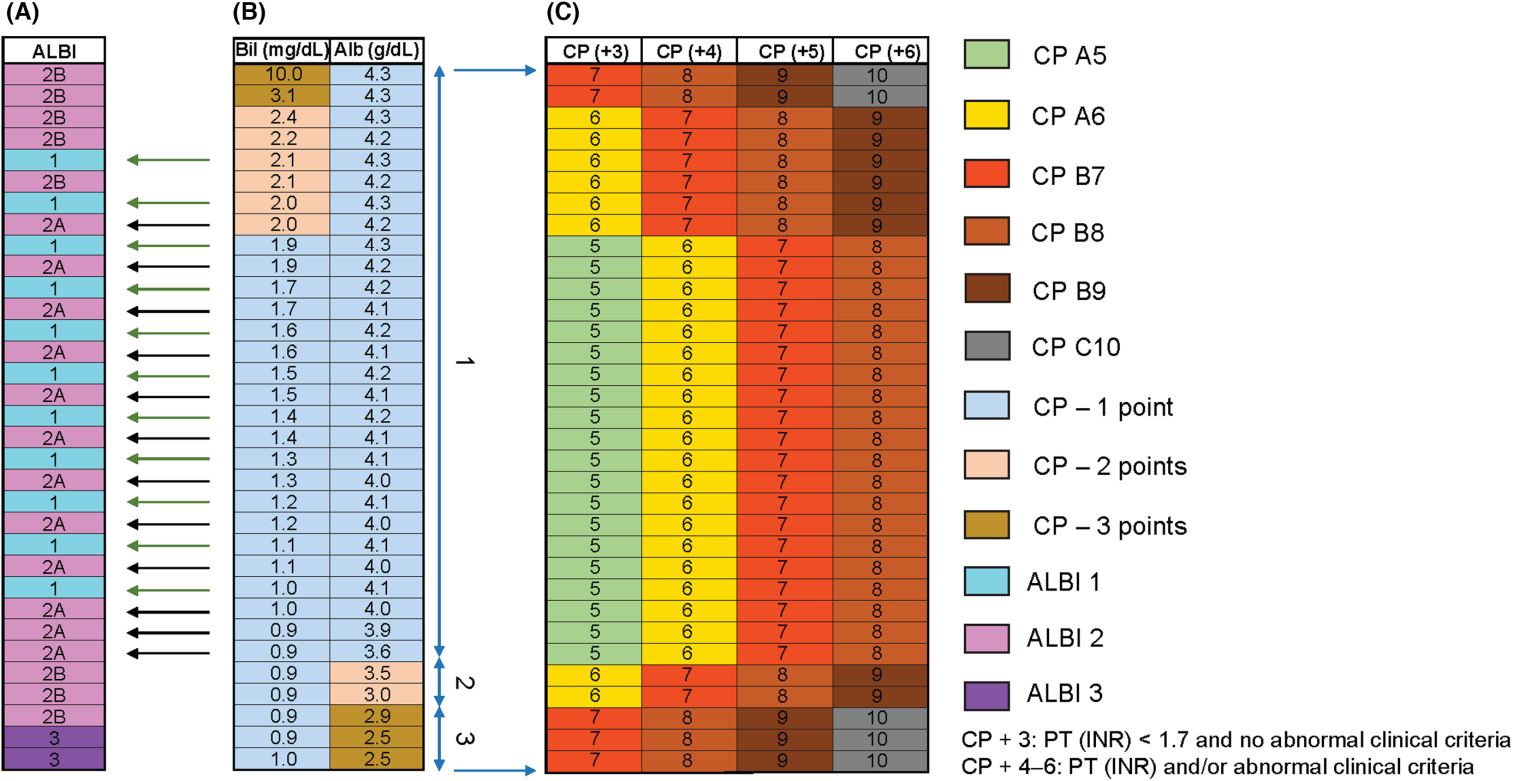
Albumin and bilirubin levels (panel B) are used in the CP classification (panel C) and ALBI grading systems (panel A) for liver dysfunction. Albumin and bilirubin levels are combined to provide an ALBI grading of 1, 2A, 2B, or 3. The CP system additionally assigns points for clinical criteria so that patients are allocated one of five classes across CP A (5–6 points) and CP B (7–9 points). The overlap in albumin and bilirubin combinations means that patients with CP A5 or A6 could be assigned to ALBI 1, 2A, or 2B. (Left column) ALBI scores were calculated to determine the boundaries between 1 and 2A; 2A and 2B; and 2B and 3 according to albumin and bilirubin values. (Right columns) CP points based on albumin and bilirubin levels are combined with points for clinical criteria (PT [INR], ascites, and encephalopathy) to provide a total score of 5–10 (CP A5, A6, B7, B8, B9, or C10).^37^ Alb, albumin; ALBI, albumin–bilirubin; Bil, bilirubin; CP, Child–Pugh; INR, international normalized ratio; PT, prothrombin.

ALBI grading has been evaluated in multiple geographical locations and clinical settings,^25^ and has been correlated with other classification systems for liver function and HCC and with outcomes in clinical trials.^28^ In a multicenter cohort study, ALBI grade was highly concordant with CP‐based classification (*n* = 3696).^29^, ^30^ The overall prognostic performance of ALBI grading is particularly good in intermediate‐ and advanced‐stage disease.^31^, ^32^, ^33^, ^34^, ^35^, ^36^ (Figure 1). Subdivision of ALBI 2 has also been proposed.^38^

New drugs for HCC are initially tested in patients with adequate liver function, but results may not translate to patients with moderate liver dysfunction.

### 1.2 Treatment landscape for advanced HCC

#### 1.2.1 Oral multikinase inhibitors (MKIs)

Several MKIs have been approved for the treatment of advanced HCC based on improvements in overall survival (OS).^20^, ^39^, ^40^, ^41^ The Phase 3 randomized controlled trials supporting these approvals primarily included patients with CP A. Efficacy data for patients with baseline ALBI 2 or CP B are typically only available from ad hoc or post hoc analyses of Phase 3 trials, prospective Phase 2 trials, and retrospective studies. Sorafenib is one exception, with Phase 3 data from patients with CP B available. The National Comprehensive Cancer Network (NCCN) Clinical Practical Guidelines in Oncology (www.nccn.org/guidelines) recommends this drug for the treatment of advanced HCC in patients with moderate liver dysfunction (i.e. CP B7).

##### Sorafenib

Sorafenib, an oral inhibitor of Raf‐1, B‐Raf, vascular endothelial growth factor receptors (VEGFRs) 1, 2, and 3, and platelet‐derived growth factor receptor (PDGFR), improved OS compared with placebo in the SHARP Phase 3 trial.^40^ In a Phase 2 study of first‐line sorafenib in patients with advanced HCC, those with CP B (*n* = 38 [28%]) fared worse than those with CP A, with 12.9 weeks therapy duration, 13 weeks median progression‐free survival (PFS), 14 weeks OS, and encephalopathy and worsening ascites in 11% and 18% of patients, respectively.^42^, ^43^ In another Phase 2 study, 14.3% of patients with baseline ALBI 2 (*n* = 162) discontinued treatment early and mortality was higher in patients with worsening liver function than in those with normal function.^44^ In a retrospective study of 300 patients with advanced HCC, 58 patients with CP B had a median OS of 3.8 months compared with 10.0 months for patients with CP A (*n* = 234).^45^

GIDEON was a prospective, observational registry study to evaluate the real‐world safety of sorafenib in patients with HCC. Data from 669 treatment‐naive patients with CP B were included.^46^ The median treatment duration of 9.9 weeks in patients with CP B was longer in those with CP B7 (11.1 weeks) than in those with CP B8 (9.3 weeks) and CP B9 (7.6 weeks). The median OS was 5.2 months in those with CP B (CP B7, 6.2 months; CP B8, 4.8 months; CP B9, 3.7 months), much shorter than in CP A (13.6 months). Patients with CP B also had higher rates of adverse events (AEs) than those with CP A (Table 1).^46^

**TABLE 1:** Toxicity reported in the GIDEON trial of first‐line sorafenib in patients with advanced HCC and Child–Pugh A or Child–Pugh B.^46^

	Child–Pugh A	Child–Pugh B
Adverse events	(*n* = 1777)	(*n* = 576)
Any	84%	88%
Drug‐related	69%	64%
Serious	36%	60%
Serious drug‐related	9%	15%
Most common adverse events	(*n* = 1968)	(*n* = 666)
Diarrhea	54%	71%
Hand‐foot syndrome	55%	42%
Fatigue	38%	62%
Adverse events leading to discontinuation	(*n* = 1968)	(*n* = 666)
Any	29%	40%
Drug‐related	17%	21%

Abbreviation: HCC, hepatocellular carcinoma.

The recommended continuous dose of sorafenib is 400 mg twice daily in patients with normal liver function.^40^ A Phase 1 study of sorafenib in patients with liver or kidney dysfunction found that those with moderate to severe liver dysfunction cannot tolerate a starting dose of 400 mg sorafenib. Elevated bilirubin was a dose‐limiting toxicity^47^ and dose adjustment was recommended for this population.^46^

##### Lenvatinib

In the REFLECT Phase 3 trial, lenvatinib, an inhibitor of VEGFRs 1, 2, and 3, fibroblast growth factor receptors 1, 2, 3, and 4, PDGFR‐α, and RET and KIT proto‐oncogenes, was noninferior to sorafenib in OS in patients with HCC.^20^ In a post hoc analysis, the median OS among patients with baseline ALBI 1 and 2 was 17.4 months and 8.6 months, respectively.^48^ In a Phase 2 study, patients with ALBI 2 had a time to treatment failure of 5.3 months and a higher rate of AE‐related treatment discontinuation than patients with ALBI 1 (*p* < 0.01).^49^ There are no Phase 3 data to support the use of lenvatinib in patients with CP B; sorafenib is often used as the first‐line treatment in this population.^50^ In a retrospective cohort study of patients receiving either sorafenib or lenvatinib as first‐line treatment, those with baseline ALBI 2B had a longer median overall treatment duration (4.5 months) than patients with baseline ALBI 3 (3.0 months).^38^

##### Regorafenib

Regorafenib is an inhibitor of oncogenic kinases including RET, RAF‐1, and KIT and appears to be more potent than sorafenib.^51^ In the RESORCE Phase 3 trial, regorafenib improved OS compared with placebo in patients previously treated with sorafenib.^39^ Patients with ALBI 2 receiving regorafenib had significantly worse OS than those with ALBI 1 (hazard ratio [HR] 0.432; *p* = 0.001).^52^ In a multicenter retrospective study in patients receiving regorafenib after sorafenib, median PFS and OS were 1.8 and 4.6 months, respectively, in the CP B cohort (*n* = 59), which was significantly worse than the CP A cohort (*p* = 0.008 and *p* < 0.001, respectively).^53^ In another multicenter retrospective study of patients with CP A receiving regorafenib after sorafenib (*N* = 440), median PFS and OS were 3.2 and 12.1 months, respectively.^54^ ALBI grade can be used to identify patients suitable for first‐ and second‐line regorafenib treatment and those at high risk of liver function deterioration with treatment, according to a retrospective cohort study of regorafenib treatment in patients with progressive disease previously treated with sorafenib.^51^, ^55^

##### Cabozantinib

Cabozantinib inhibits tyrosine kinases, including VEGFRs 1–3, MET, and AXL, and was approved based on the CELESTIAL Phase 3 trial.^41^ In a post hoc analysis of CELESTIAL, 51 patients receiving cabozantinib were classified as CP B at week 8 of follow‐up. The median OS was 3.8 months for patients with CP B, shorter than in patients with CP A (HR 0.32, 95% CI 0.18, −0.58).^56^ Patients receiving cabozantinib after progression on other systemic therapies were stratified by CP class at time of cabozantinib initiation in a multicenter retrospective study. CP B was found to be independently prognostic of worse PFS (HR 3.38, 95% CI 1.50–4.60; *p* = 0.001) and OS (HR 4.95, 95% CI 2.81–10.2; *p* < 0.001) when compared with CP A. The median PFS and OS were 2.2 and 3.8 months, respectively, in patients with CP B (*n* = 22), compared with 4.3 (*p* < 0.001) and 9.0 (*p* < 0.001) months, respectively, in patients with CP A (*n* = 88).^57^

#### 1.2.2 Immuno‐oncology (IO) agents

For many years, sorafenib was the only systemic treatment for advanced HCC with proven efficacy.^58^ Since 2018, promising IO agents have been tested in patients with HCC. These include monoclonal antibodies targeting VEGFRs and immune checkpoint inhibitors (ICIs), such as monoclonal antibodies targeting programmed cell death protein 1 (PD‐1), programmed cell death ligand 1 (PD‐L1), and cytotoxic T‐lymphocyte‐associated antigen 4 (CTLA‐4). The meaningful improvements in OS, high objective response rates, and favorable toxicity profiles of IO agents versus sorafenib in patients with advanced HCC supported approval^59^, ^60^ for IO combinations in first‐line therapy, restricted to patients with good liver function (CP A), no active virus infection, and low risk of bleeding.^16^, ^17^

##### Anti‐VEGF monotherapy with ramucirumab

In the REACH Phase 3 trial, ramucirumab, a recombinant immunoglobulin G1 monoclonal antibody that binds to VEGFR‐2, failed to demonstrate improvement against placebo in OS in patients with advanced HCC previously treated with sorafenib.^61^ The original protocol included patients with CP B, but a protocol amendment later excluded these patients from the analysis. In an exploratory analysis of 78 patients with CP B7–8 at baseline, there was no difference in median OS between the treatment arms (HR 1.00, 95% CI 0.62, 1.60; *p* ≥ 0.99) and there was a higher incidence of Grade 3 or 4 toxicity than in patients with CP A.^62^ Stratification of median OS by ALBI in the REFLECT, RESORCE, CELESTIAL, and REACH trials showed that MKIs and ramucirumab were no better than sorafenib in patients with advanced HCC and moderate liver dysfunction.

##### ICI monotherapy

The single‐agent PD‐1 inhibitors nivolumab^63^ and pembrolizumab^64^ received accelerated approval for use in patients with advanced HCC after sorafenib based on the Phase 1/2 CheckMate 040^65^ and Phase 2 KEYNOTE‐224^66^ trials, respectively. The CheckMate 040 trial of nivolumab enrolled a small number of patients (*n* = 49) with CP B7–8; median OS for nivolumab was 7.6 months in this cohort, compared with a historical OS of 2.5–5.4 months for patients with CP A treated with sorafenib.^65^ Nivolumab and pembrolizumab later failed to improve OS versus sorafenib (nivolumab) or placebo (pembrolizumab) in Phase 3 trials.^18^, ^67^

###### ICI and anti‐VEGF combination therapy

ICIs, in combination or when combined with anti‐VEGF agents, have shown synergistic antitumor effects and better quality of life (QOL) endpoints compared with sorafenib in patients with advanced HCC and CP A.^23^ First‐line atezolizumab plus bevacizumab was proven to be superior to sorafenib (HR 0.58; *p* < 0.001) in the Phase 3 IMbrave150 trial in patients with advanced HCC and CP A who had not previously received any systemic treatment.^16^, ^68^ After an additional 12 months of follow‐up of IMbrave150, atezolizumab plus bevacizumab maintained a clinically meaningful survival benefit over sorafenib and had a safety profile consistent with the primary analysis.^68^ This combination is now standard of care for first‐line treatment for advanced HCC in patients with good liver function.^69^

When stratified by ALBI, the IMbrave150 trial found no difference in median OS between atezolizumab plus bevacizumab (11.7 months) and sorafenib (12.2 months) in patients with ALBI 2.^68^ In a separate retrospective real‐world study of 147 patients treated with atezolizumab plus bevacizumab, 73 patients would have been excluded from the IMbrave150 trial based on at least one major exclusion criterion. Median OS was 6.0 months for IMbrave150‐ineligible patients, and was significantly longer in IMbrave150‐eligible patients (*p* < 0.001), while median OS in patients with ALBI 2 and 3 was 8.6 months and 3.2 months, respectively.^70^ In a real‐world retrospective study of 216 patients with HCC treated consecutively with atezolizumab and bevacizumab, 154 patients with CP A had a median OS of 16.8 months versus 6.7 months for 48 patients with CP B (*p* = 0.0003).^71^

###### Other ICI combination therapies

Tremelimumab plus durvalumab also showed superiority over sorafenib as first‐line therapy in patients with advanced HCC and CP A in the Phase 3 HIMALAYA trial.^17^ These data demonstrate the importance of PD‐L1 and CTLA‐4 in the cellular immune response to HCC.

Several studies combining IO agents with MKIs and other biological agents in first‐ and second‐line therapy are ongoing. A complete list of studies can be found in a recent review.^23^

##### Remaining challenges in immunotherapy for HCC

Despite the advances in IO agents for treating advanced HCC, several challenges remain. Phase 3 trials of IO agents in HCC have typically been restricted to a group of patients with good liver function (CP A) and their use in patients with moderate liver dysfunction has not been fully evaluated. Owing to a lack of available therapies, patients with some residual liver function (CP B7) may receive prescriptions for IO agents, even though supporting data are restricted to CP A and there are few or no data for CP B.

Even among patients with advanced HCC and good liver function, there remains a need for validated biomarkers for predicting and assessing responses to IO agents^72^; up to 70% of patients with advanced HCC treated with ICIs may not benefit from immunotherapy.^73^ PD‐L1 expression, tumor mutational burden, microsatellite instability status, and gut microbiota are among the biomarkers being assessed.^73^ Combining ICIs with non‐ICI treatments in first‐line therapy might be beneficial for reducing the risk of early death associated with the use of ICIs alone;^74^ one suggested combination is an ICI, an anti‐VEGF agent, and ^90^Yttrium transarterial radioembolization.^75^ Such strategies should be further explored.

### 1.3 Complications of liver disease

Cirrhosis represents a late stage of progressive hepatic fibrosis and is generally considered irreversible in its advanced stages. Cirrhosis accounted for approximately 50,000 deaths in the US in 2010 and was the eighth leading cause of death.^76^ There are several complications associated with cirrhosis, and patients can have a significantly reduced life expectancy.^77^ This creates challenges in the design and conduct of prospective clinical trials in HCC because the confounding risk of cirrhosis can bias evaluation of the efficacy of anticancer therapy. Moreover, reducing tumor burden does not necessarily lead to improvements in liver function and survival.

Risk factors for cirrhosis complications include bleeding, infection, alcohol consumption, medication use, dehydration, constipation, and obesity.^78^, ^79^, ^80^, ^81^ Many of the complications of cirrhosis are associated with high mortality. Variceal hemorrhage is associated with a 30‐day mortality of 15%–30%, despite improvements in techniques for controlling bleeding.^82^ Portal hypertensive gastropathy (congestive gastropathy), a common complication in patients with portal hypertension, can also cause gastrointestinal bleeding and anemia.^83^ Patients with cirrhosis are at increased risk of developing portal vein thrombosis, which in turn increases the risk of portal hypertension.^84^ Ascites is another common, major complication of cirrhosis and can lead to patients requiring repeated therapeutic paracenteses or placement of a transjugular intrahepatic portosystemic shunt.^85^

Spontaneous bacterial peritonitis is common in patients with end‐stage liver disease and is associated with high mortality without early antibiotic treatment.^86^ Complications of cirrhosis can also involve other organs. Progressive hepatic injury leading to reductions in renal perfusion can culminate in hepatorenal syndrome. Without an improvement in hepatic function or liver transplantation the prognosis of hepatorenal syndrome is poor.^87^ Estimates of the prevalence of hepatopulmonary syndrome range from 4% to 47% in patients with cirrhosis, depending on the diagnostic criteria and methods used. There are no effective medical therapies for hepatopulmonary syndrome and it must be excluded in clinical trials if radioembolization is planned.^88^ Other complications include cardiac dysfunction, which is observed in up to half of patients with advanced cirrhosis undergoing liver transplantation,^89^ and hepatic encephalopathy.^90^

### 1.4 Clinical development in patients with moderate liver dysfunction

**FIGURE 2:**
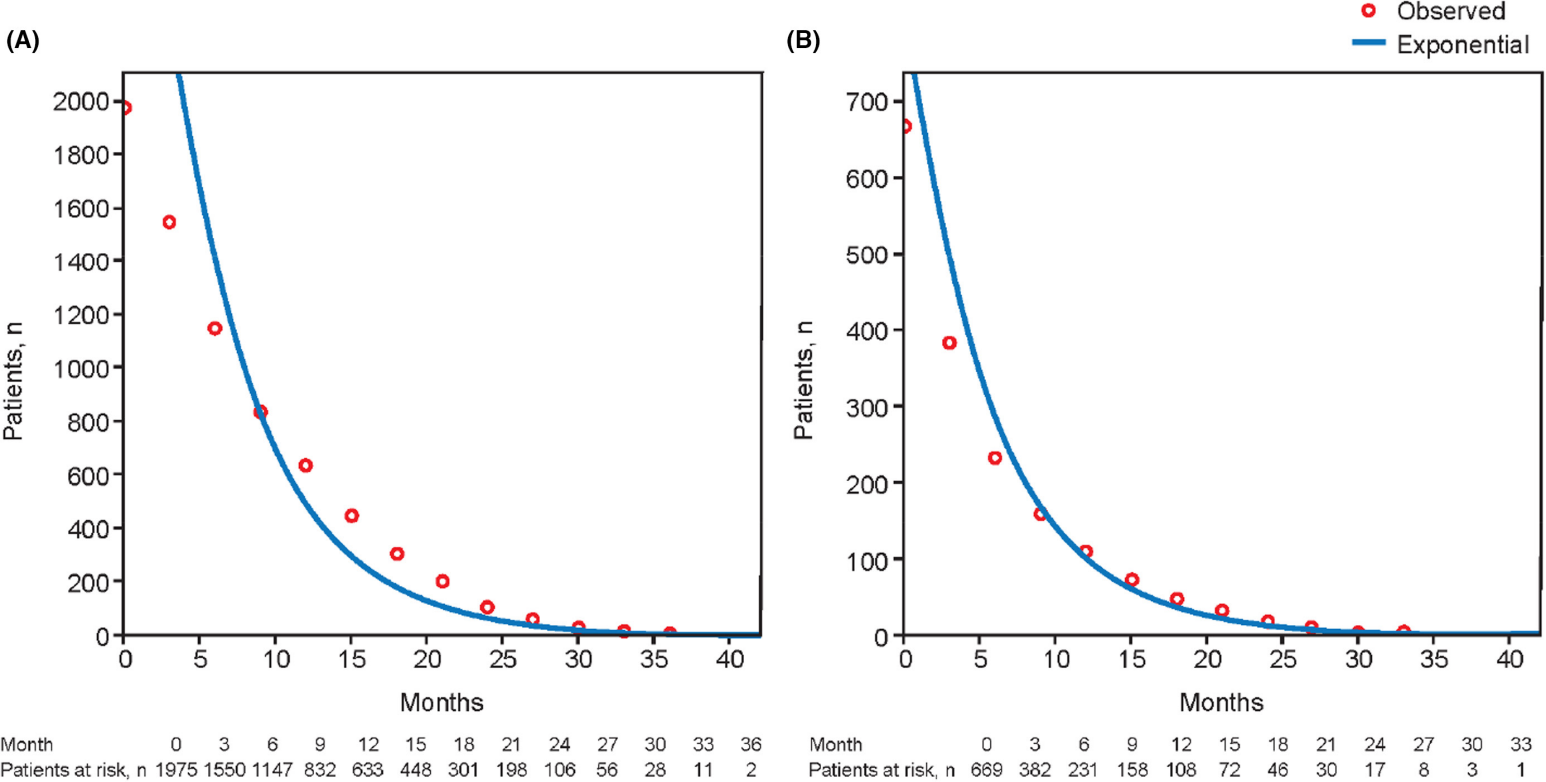
Exponential survival distribution of censored treatment‐naive patients with (A) Child–Pugh A and (B) Child–Pugh B (GIDEON study).^46^

Complications of cirrhosis limit clinical development of treatments for HCC in patients with moderate liver dysfunction. Heterogeneity of the population and the shorter life expectancy and increased risk of sudden death events in those with CP B versus CP A limit the ability to assess efficacy in prospective clinical trials. Hepatic deterioration during treatment is also observed in patients with moderate liver dysfunction. This could be related to toxicity of treatment or progression of the tumor burden resulting in intrahepatic obstruction of the biliary tree and/or portal vein.^11^, ^91^, ^92^ The unpredictable events associated with moderate liver dysfunction led to a different survival distribution among patients with CP B and those with CP A. In the GIDEON study, the survival distribution of treatment‐naive patients with CP B treated with sorafenib could be modeled using an exponential distribution function (Figure 2).^46^

**FIGURE 3:**
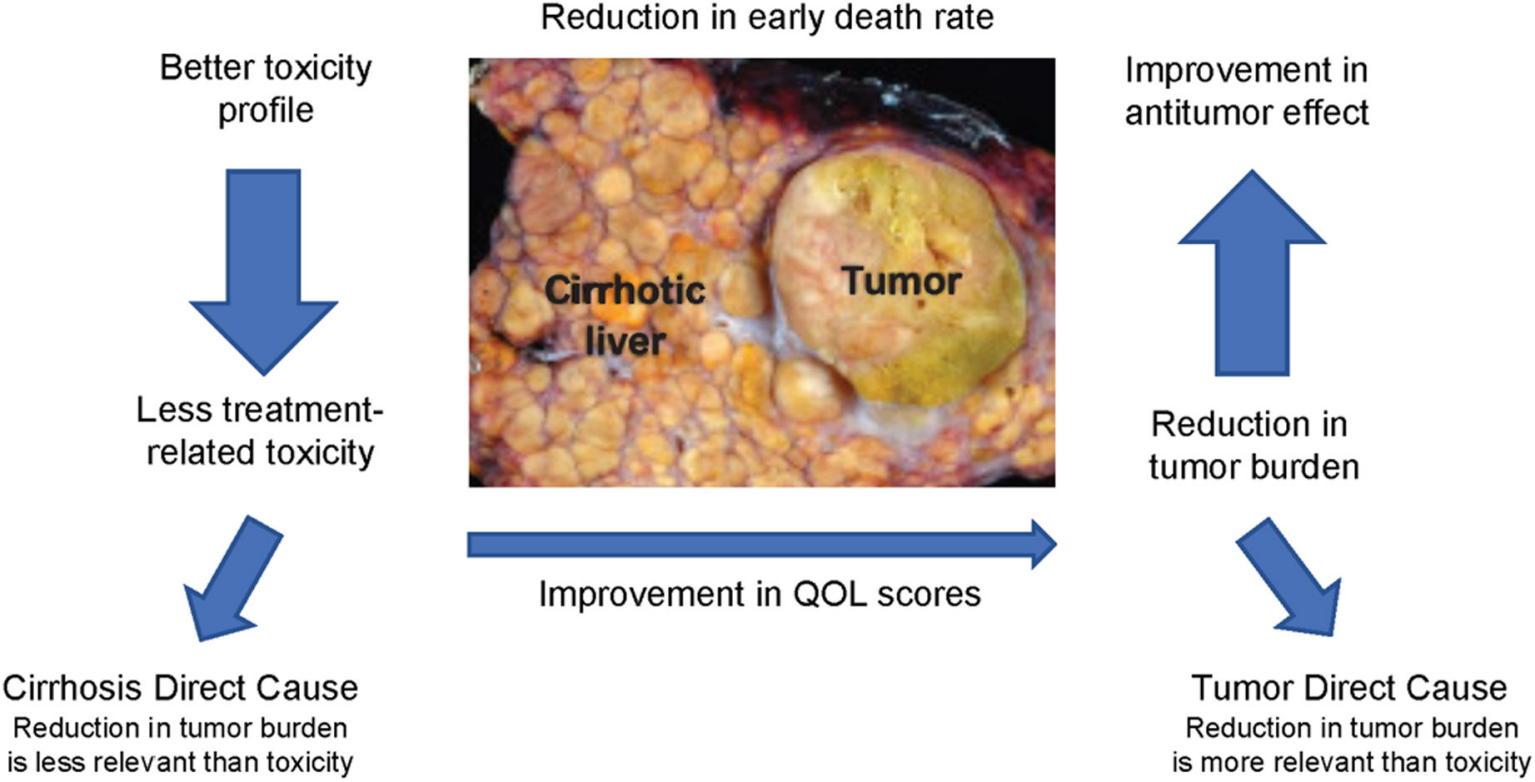
Schematic approach for ideal treatment intervention in patients with HCC and moderate liver dysfunction. HCC, hepatocellular carcinoma; QOL, quality of life.

Interventions involving a balanced approach to treatment of both cirrhosis and tumor burden would likely improve the long‐term survival and QOL of patients with HCC and moderate liver dysfunction (Figure 3).

### 1.5 Endpoints in clinical trials of HCC treatments in patients with moderate liver dysfunction

The optimal efficacy endpoint for clinical trials in patients with HCC and moderate liver dysfunction is a topic for consideration. OS is objective and clinically relevant and is the usual primary endpoint used in randomized Phase 3 clinical trials. OS is therefore the basis of most regulatory approval processes for drugs and other treatment interventions. However, OS is likely to be disproportionately affected by early death rates in patients with moderate liver dysfunction compared with those with normal liver function.^93^ Early death (death occurring within 3 months from the date of diagnosis) has been observed in patients with HCC and severe cirrhosis. A recent study in the Surveillance, Epidemiology, and End Results database reported that 44.6% of patients with tumor, node, metastases (TNM) stage IVA and 62.8% of patients with TNM stage IVB HCC experienced early death.^94^

**FIGURE 4:**
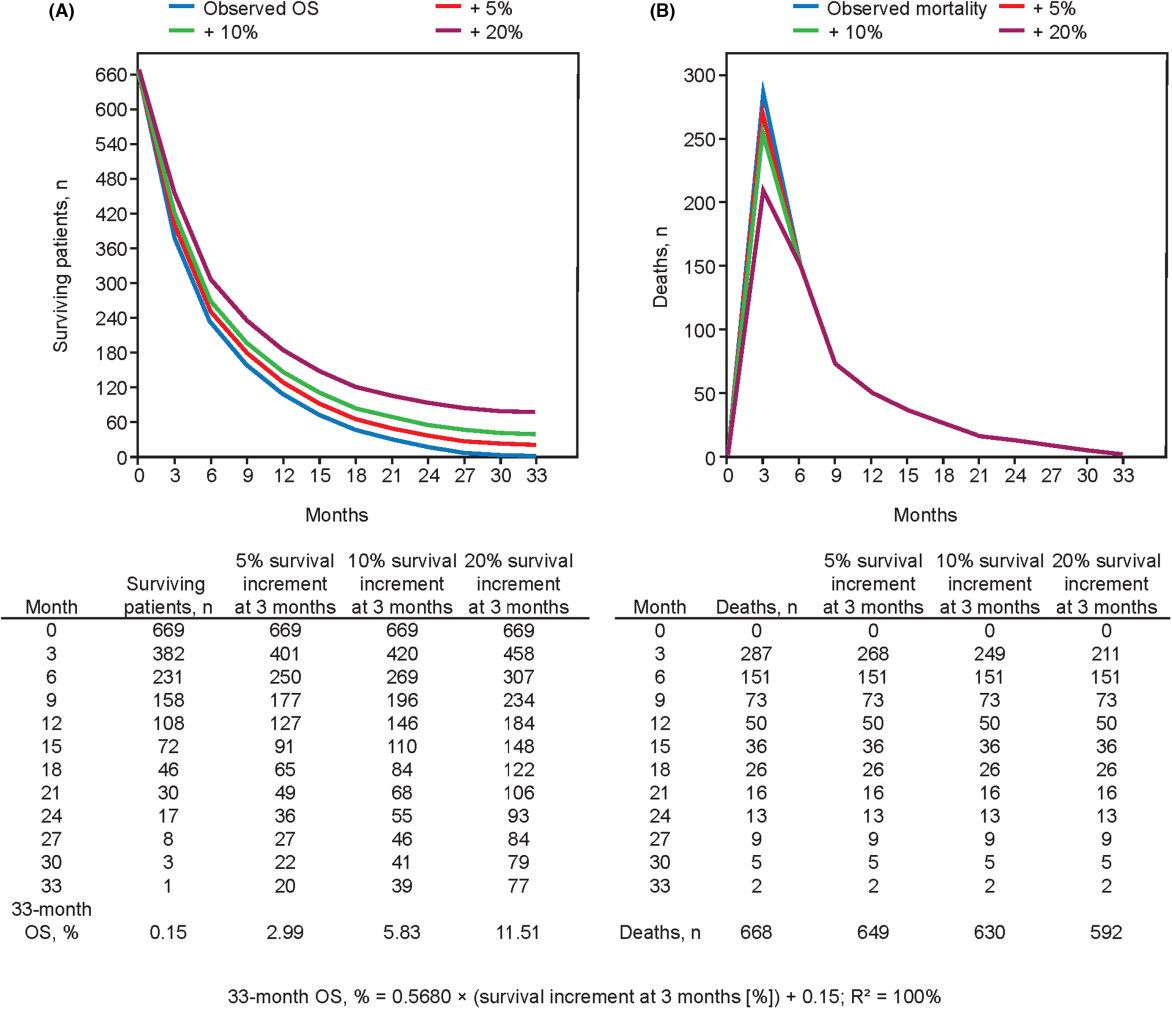
Simulations of outcomes for a treatment intervention resulting in 3‐month survival increment of 5%, 10%, or 20%, showing (A) a linear relationship with 33‐month OS and (B) reduction of early deaths in the first 3 months of follow‐up. OS, overall survival.

Simulations of incremental treatment‐related improvements in 3‐month OS for patients with CP B predict meaningful improvements in long‐term OS, suggesting that prevention of early death may represent an improved efficacy outcome (Figure 4). The main surrogate endpoints in oncology such as PFS, time to progression, and objective response rate could also be affected by early death in patients with CP B, although these endpoints seem to correlate with median OS similarly in those with CP B and those with CP A.^56^, ^95^

**FIGURE 5:**
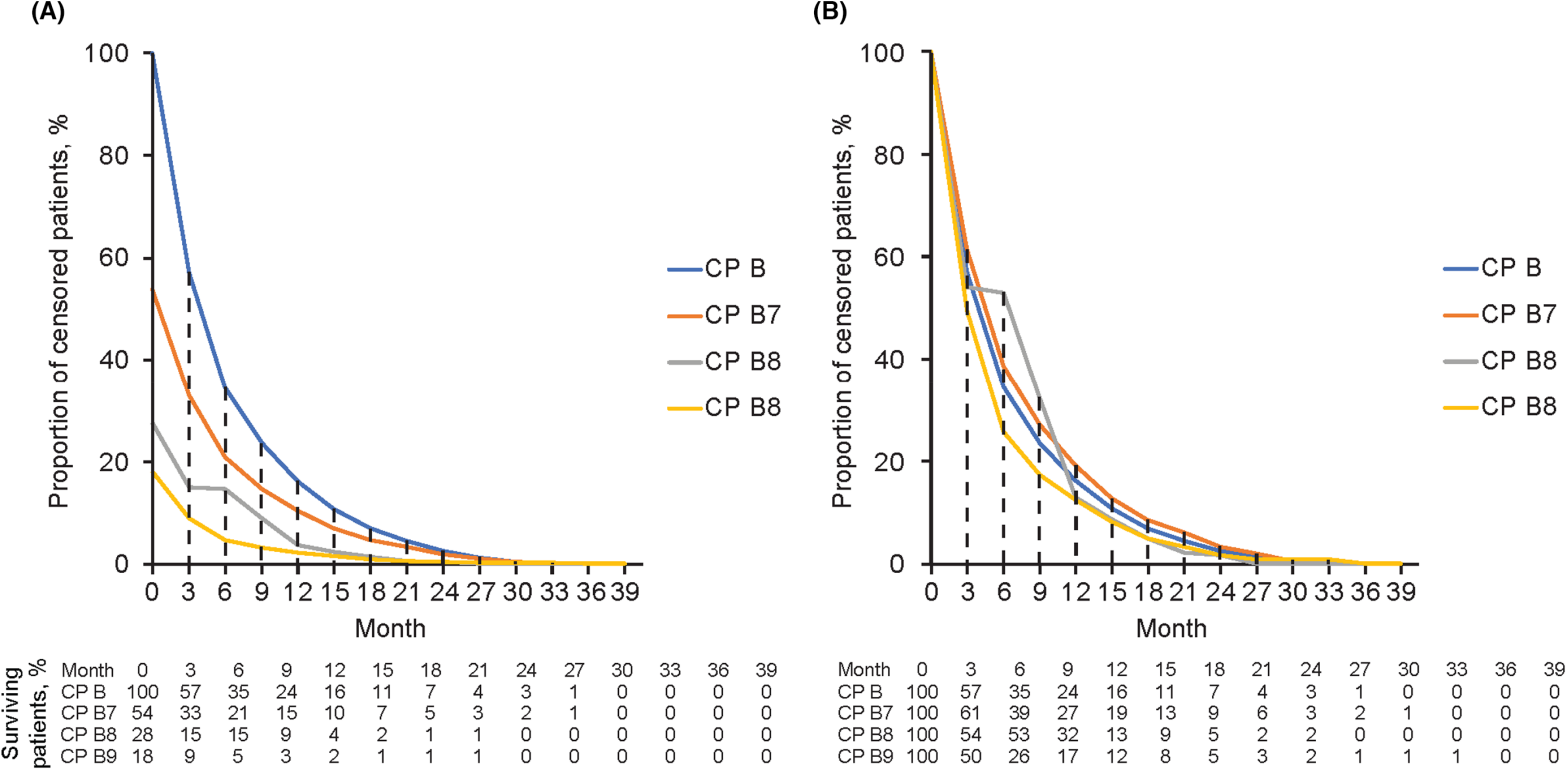
(A) Relative and (B) absolute percentage of surviving patients by month of follow‐up based on treatment‐naive patients with CP B (*n* = 669, stratified by CP B7–9) treated with sorafenib in the GIDEON study.^46^ CP, Child–Pugh.

Apart from CP classification and comorbidities, other clinical characteristics and tumor‐related variables may contribute to early death rates.^94^ Detailed assessment of treatment‐related toxicity is vital because safety is especially relevant for this population of patients with moderate liver dysfunction. HCC clinical trials require that patients are assessed for radiological response every 6–8 weeks.^93^ Investigators should consider that a significant proportion of patients may die before radiological assessment, especially when toxic interventions are studied prospectively in patients with severe cirrhosis. Figure 5 shows that nearly half of the patients with CP B had died at 3‐month follow‐up in the GIDEON study.

These uncertainties in interpreting clinical trial results mean that it is difficult to evaluate whether patients with CP B are likely to achieve similar benefits from treatment as patients with CP A.

#### 1.5.1 Clinical trials including patients with moderate liver dysfunction

A few prospective systemic treatment clinical trials including patients with CP B are ongoing, two of which restrict eligibility to patients with CP B only. Cabozantinib is being tested in a Phase 1/2 study as a first‐ and second‐line therapy in 32 patients (NCT04497038) and 20 patients (NCT04454762) with CP B7–8 and ECOG PS 0–1. The primary endpoint is maximum tolerated dose for a Phase 2 study and the secondary endpoints are PFS, OS, and median time to progression. Atezolizumab and bevacizumab are being tested in a Phase 2 study as first‐line therapies in 50 patients with CP B7 and ECOG PS 0–1 (NCT04829383). The primary endpoint is frequency and severity of toxicities, and the secondary endpoints are overall response, disease control rate, duration of response, OS, and PFS. The other study lists CP B7 or CP A as inclusion criteria (NCT03439891).

### 1.6 Impact of chronic liver disease on QOL

Ongoing symptoms, treatment‐related issues such as AEs and disease progression, and other emotional, physical, and social factors such as anxiety, fatigue, and stigmatization, all negatively impact QOL in patients with chronic liver diseases.^96^ The National Cancer Institute's Patient‐Reported Outcomes version of the Common Terminology Criteria for AEs provides a standard method to assess symptomatic AEs from the perspective of the physician.^97^ Systematic measurement of QOL using patient‐reported outcomes is a standard method and enables comparison of outcomes between treatment arms (including placebo). The European Organization for Research and Treatment of Cancer Quality of Life Questionnaire and the Functional Assessment of Cancer Therapy‐Hepatobiliary questionnaire are two established methods used in HCC clinical trials.^98^, ^99^, ^100^ These data can inform and support the development of new therapies in HCC. Patients experiencing maintained QOL scores should generally also have improvement in OS;^101^, ^102^ however, this correlation still needs to be shown in patients with CP B. Sequential measurement using QOL questionnaires can be a sensitive procedure to identify early benefits of a treatment intervention. Time to deterioration in QOL may correlate with median OS and long‐term survival, and can be combined with other efficacy endpoints such as PFS and OS in patients with CP B.

### 1.7 Future directions

Effective treatment of patients with advanced HCC and CP B is an important unmet need. New treatment strategies are promising based on demonstrated activity and tolerability in patients with advanced HCC, including IO agents alone, in combination with existing therapies, or in combination with new targeted agents such as MKIs. However, for patients with moderate liver dysfunction, avoiding further impairment of liver function and maintaining QOL are important considerations for the development of treatments for HCC. Treatments targeting multiple immune mechanisms appear to be especially promising in CP B, although a better understanding of the mechanisms of action at molecular and cellular levels is needed. Validated biomarkers could guide the development of IO agents in patients with HCC and CP B. The use of medical devices, such as tumor‐treating fields,^103^ as well as adoptive cell transfer, vaccination, and virotherapy represent possible new treatments to be studied in this patient population.

### 1.8 Limitations of this review

This paper is not a systematic literature review and was not performed in accordance with the Preferred Reporting Items for Systematic Reviews and Meta‐Analyses (PRISMA) guidelines. The quality of evidence in the included articles was not assessed. The main limitation of this review is that it may be subject to selection and confirmation bias. In addition, the review may have been limited by the availability and discovery of relevant data, as well as publication bias.

## 2 CONCLUSIONS

There are insufficient data on the efficacy of treatments for advanced HCC in patients with moderate liver dysfunction (specifically CP B). This is a neglected subgroup of patients and given the high frequency of liver dysfunction in patients with HCC there is a significant need for research into the efficacy and safety of new and existing treatments in this population. Sorafenib is the only available therapy for HCC with reasonable efficacy and safety data collected in patients with moderate liver dysfunction. Studies comparing sorafenib and newer treatment alternatives have yielded similar results, but systemic treatment in patients with moderate liver dysfunction is associated with high toxicity and early death rates. New prospective clinical trials focused on the CP B population are urgently needed, with study designs that consider the impact of both liver disease and tumor burden. Understanding the risk characteristics of this patient population and what is considered a meaningful clinical outcome is fundamental in the design of new clinical trials.

## AUTHOR CONTRIBUTIONS

**Frederico P. Costa:** Writing—original draft (lead). **Bertram Wiedenmann:** Writing—original draft (equal). **Christoph Roderburg:** Writing—review and editing (equal). **Raphael Mohr:** Writing—review and editing (equal). **Ghassan K. Abou‐Alfa:** Writing—review and editing (lead).

## FUNDING INFORMATION

This work was funded by Autem Therapeutics.

## CONFLICT OF INTEREST STATEMENT

Frederico Costa and Bertram Wiedenmann are employees and shareholders of Autem Therapeutics. Ghassan K. Abou‐Alfa reports institutional research support from Arcus Biosciences, AstraZeneca, BioNtech, Bristol‐Myers Squibb, Celgene, Flatiron Health, Genentech/Roche, Genoscience Pharma, Incyte, Polaris Pharmaceuticals, Puma Biotechnology, QED Therapeutics, Silenseed, and Yiviva, and consulting support from Adicet Bio, Alnylam Pharmaceuticals, AstraZeneca, Autem Therapeutics, BeiGene, Berry Genomics, Boehringer Ingelheim, Celgene, Cend Therapeutics, CytomX Therapeutics, Eisai, Eli Lilly and Company, Exelixis, Flatiron Health, Genentech/Roche, Genoscience Pharma, Helio Health, Helsinn Healthcare, Incyte, Ipsen, Merck, Nerviano Medical Sciences, NewBridge Pharmaceuticals, Novartis, QED Therapeutics, Rafael Pharmaceuticals, RedHill Biopharma, Servier, Silenseed, Sobi, Vector BioPharma, and Yiviva. The remaining authors made no disclosures.

## Data Availability

Data sharing is not applicable to this article as no new data were created or analyzed in this study.

## References

[1] Asrani AK , Devarbhavi H , Eaton J , Kamath PS . Burden of liver diseases in the world. J Hepatol. 2019;70(1):151‐171. 30266282 10.1016/j.jhep.2018.09.014

[2] Cholankeril G , Patel R , Khurana S , Satapathy SK . Hepatocellular carcinoma in non‐alcoholic steatohepatitis: current knowledge and implications for management. World J Hepatol. 2017;9(11):533‐543. 28469809 10.4254/wjh.v9.i11.533 PMC5395802

[3] Zamor PJ , deLemos AS , Russo MW . Viral hepatitis and hepatocellular carcinoma: etiology and management. J Gastrointest Oncol. 2017;8(2):229‐242. 28480063 10.21037/jgo.2017.03.14 PMC5401856

[4] Iida‐Ueno A , Enomoto M , Tamori A , Kawada N . Hepatitis B virus infection and alcohol consumption. World J Gastroenterol. 2017;23(15):2651‐2659. 28487602 10.3748/wjg.v23.i15.2651 PMC5403744

[5] McGlynn KA , London WT . The global epidemiology of hepatocellular carcinoma: present and future. Clin Liver Dis. 2011;15(2):223‐243. vii‐x. 21689610 10.1016/j.cld.2011.03.006 PMC4141529

[6] Ponziani FR , Mangiola F , Binda C , et al. Future of liver disease in the era of direct acting antivirals for the treatment of hepatitis C. World J Hepatol. 2017;9(7):352‐367. 28321272 10.4254/wjh.v9.i7.352 PMC5340991

[7] Llovet JM , Bru C , Bruix J . Prognosis of hepatocellular carcinoma: the BCLC staging classification. Semin Liver Dis. 1999;19(3):329‐338. 10518312 10.1055/s-2007-1007122

[8] Llovet JM , Fuster J , Bruix J , Barcelona‐Clínic Liver Cancer Group . The Barcelona approach: diagnosis, staging, and treatment of hepatocellular carcinoma. Liver Transpl. 2004;10(2 Suppl 1):S115‐S120. 14762851 10.1002/lt.20034

[9] Ferlay J , Soerjomataram I , Dikshit R , et al. Cancer incidence and mortality worldwide: sources, methods and major patterns in GLOBOCAN 2012. Int J Cancer. 2015;136(5):E359‐E386. 25220842 10.1002/ijc.29210

[10] Mittal S , El‐Serag HB . Epidemiology of hepatocellular carcinoma: consider the population. J Clin Gastroenterol. 2013;47:S2‐S6. 23632345 10.1097/MCG.0b013e3182872f29 PMC3683119

[11] Schlichting P , Christensen E , Fauerholdt L , Poulsen H , Juhl E , Tygstrup N . Main causes of death in cirrhosis. Scand J Gastroenterol. 1983;18(7):881‐888. 6374868 10.3109/00365528309182110

[12] El‐Serag HB , Davila JA . Surveillance for hepatocellular carcinoma: in whom and how? Therap Adv Gastroenterol. 2011;4(1):5‐10. 10.1177/1756283X10385964 PMC3036965 21317990

[13] Okuda H . Hepatocellular carcinoma development in cirrhosis. Best Pract Res Clin Gastroenterol. 2007;21(1):161‐173. 17223503 10.1016/j.bpg.2006.07.002

[14] Bruix J , Han KH , Gores G , Llovet JM , Mazzaferro V . Liver cancer: approaching a personalized care. J Hepatol. 2015;62(1 Suppl):S144‐S156. 25920083 10.1016/j.jhep.2015.02.007 PMC4520430

[15] Llovet JM , Zucman‐Rossi J , Pikarsky E , et al. Hepatocellular carcinoma. Nat Rev Dis Primers. 2016;2:16018. 27158749 10.1038/nrdp.2016.18

[16] Finn RS , Qin S , Ikeda M , et al. Atezolizumab plus bevacizumab in unresectable hepatocellular carcinoma. N Engl J Med. 2020;382(20):1894‐1905. 32402160 10.1056/NEJMoa1915745

[17] Abou‐Alfa Ghassan K , Lau G , Kudo M , et al. Tremelimumab plus durvalumab in unresectable hepatocellular carcinoma. NEJM Evidence. 2022;1(8):EVIDoa2100070. 10.1056/EVIDoa2100070 38319892

[18] Yau T , Park JW , Finn RS , et al. Nivolumab versus sorafenib in advanced hepatocellular carcinoma (CheckMate 459): a randomised, multicentre, open‐label, phase 3 trial. Lancet Oncol. 2022;23(1):77‐90. 34914889 10.1016/S1470-2045(21)00604-5

[19] Kelley RK , Rimassa L , Cheng AL , et al. Cabozantinib plus atezolizumab versus sorafenib for advanced hepatocellular carcinoma (COSMIC‐312): a multicentre, open‐label, randomised, phase 3 trial. Lancet Oncol. 2022;23(8):995‐1008. 35798016 10.1016/S1470-2045(22)00326-6

[20] Kudo M , Finn RS , Qin S , et al. Lenvatinib versus sorafenib in first‐line treatment of patients with unresectable hepatocellular carcinoma: a randomised phase 3 non‐inferiority trial. Lancet. 2018;391(10126):1163‐1173. 29433850 10.1016/S0140-6736(18)30207-1

[21] Finn RS , Kudo M , Merle P , et al. Primary results from the phase III LEAP‐002 study: lenvatinib plus pembrolizumab versus lenvatinib as first‐line (1L) therapy for advanced hepatocellular carcinoma (aHCC). Ann Oncol. 2022;33:S808‐S869.

[22] Reig M , Forner A , Rimola J , et al. BCLC strategy for prognosis prediction and treatment recommendation: the 2022 update. J Hepatol. 2022;76(3):681‐693. 34801630 10.1016/j.jhep.2021.11.018 PMC8866082

[23] Bejjani AC , Finn RS . Hepatocellular carcinoma: pick the winner‐tyrosine kinase inhibitor versus immuno‐oncology agent‐based combinations. J Clin Oncol. 2022;40(24):2763‐2773. 35649192 10.1200/JCO.21.02605

[24] Rimassa L , Personeni N , Czauderna C , Foerster F , Galle P . Systemic treatment of HCC in special populations. J Hepatol. 2021;74(4):931‐943. 33248171 10.1016/j.jhep.2020.11.026

[25] Johnson PJ , Berhane S , Kagebayashi C , et al. Assessment of liver function in patients with hepatocellular carcinoma: a new evidence‐based approach‐the ALBI grade. J Clin Oncol. 2015;33(6):550‐558. 25512453 10.1200/JCO.2014.57.9151 PMC4322258

[26] Kudo M , Arizumi T , Ueshima K , Sakurai T , Kitano M , Nishida N . Subclassification of BCLC B stage hepatocellular carcinoma and treatment strategies: proposal of modified Bolondi's subclassification (Kinki criteria). Dig Dis. 2015;33(6):751‐758. 26488473 10.1159/000439290

[27] D'Amico G , Garcia‐Tsao G , Pagliaro L . Natural history and prognostic indicators of survival in cirrhosis: a systematic review of 118 studies. J Hepatol. 2006;44(1):217‐231. 16298014 10.1016/j.jhep.2005.10.013

[28] Hiraoka A , Kumada T , Michitaka K , Kudo M . Newly proposed ALBI grade and ALBI‐T score as tools for assessment of hepatic function and prognosis in hepatocellular carcinoma patients. Liver Cancer. 2019;8(5):312‐325. 31768342 10.1159/000494844 PMC6873026

[29] Chan AW , Chong CC , Mo FK , et al. Applicability of albumin‐bilirubin‐based Japan integrated staging score in hepatitis B‐associated hepatocellular carcinoma. J Gastroenterol Hepatol. 2016;31(10):1766‐1772. 26992142 10.1111/jgh.13339

[30] Pinato DJ , Kaneko T , Saeed A , et al. Immunotherapy in hepatocellular cancer patients with mild to severe liver dysfunction: adjunctive role of the ALBI grade. Cancers (Basel). 2020;12(7):1862. 32664319 10.3390/cancers12071862 PMC7408648

[31] Kuzuya T , Ishigami M , Ito T , et al. Clinical characteristics and outcomes of candidates for second‐line therapy, including regorafenib and ramucirumab, for advanced hepatocellular carcinoma after sorafenib treatment. Hepatol Res. 2019;49(9):1054‐1065. 31033165 10.1111/hepr.13358

[32] Huitzil‐Melendez FD , Capanu M , O'Reilly EM , et al. Advanced hepatocellular carcinoma: which staging systems best predict prognosis? J Clin Oncol. 2010;28(17):2889‐2895. 20458042 10.1200/JCO.2009.25.9895 PMC3651603

[33] Collette S , Bonnetain F , Paoletti X , et al. Prognosis of advanced hepatocellular carcinoma: comparison of three staging systems in two French clinical trials. Ann Oncol. 2008;19(6):1117‐1126. 18303031 10.1093/annonc/mdn030

[34] Adhoute X , Penaranda G , Raoul JL , et al. Prognosis of advanced hepatocellular carcinoma: a new stratification of Barcelona Clinic Liver Cancer stage C: results from a French multicenter study. Eur J Gastroenterol Hepatol. 2016;28(4):433‐440. 26695429 10.1097/MEG.0000000000000558

[35] Yau T , Yao TJ , Chan P , Ng K , Fan ST , Poon RT . A new prognostic score system in patients with advanced hepatocellular carcinoma not amendable to locoregional therapy: implication for patient selection in systemic therapy trials. Cancer. 2008;113(10):2742‐2751. 18853421 10.1002/cncr.23878

[36] Samawi HH , Sim HW , Chan KK , et al. Prognosis of patients with hepatocellular carcinoma treated with sorafenib: a comparison of five models in a large Canadian database. Cancer Med. 2018;7:2816‐2825. 29766659 10.1002/cam4.1493 PMC6051235

[37] Hiraoka A , Michitaka K , Kumada T , et al. Validation and potential of albumin‐bilirubin grade and prognostication in a nationwide survey of 46,681 hepatocellular carcinoma patients in Japan: the need for a more detailed evaluation of hepatic function. Liver Cancer. 2017;6(4):325‐336. 29234636 10.1159/000479984 PMC5704689

[38] Hiraoka A , Tanizawa Y , Huang YJ , Cai Z , Sakaguchi S . Association of albumin‐bilirubin grade and sequential treatment with standard systemic therapies for advanced hepatocellular carcinoma: a retrospective cohort study using a Japanese administrative database. Drugs Real World Outcomes. 2021;8(3):301‐314. 33792850 10.1007/s40801-021-00245-8 PMC8324688

[39] Bruix J , Qin S , Merle P , et al. Regorafenib for patients with hepatocellular carcinoma who progressed on sorafenib treatment (RESORCE): a randomised, double‐blind, placebo‐controlled, phase 3 trial. Lancet. 2017;389(10064):56‐66. 27932229 10.1016/S0140-6736(16)32453-9

[40] Llovet JM , Ricci S , Mazzaferro V , et al. Sorafenib in advanced hepatocellular carcinoma. N Engl J Med. 2008;359(4):378‐390. 18650514 10.1056/NEJMoa0708857

[41] Abou‐Alfa GK , Meyer T , Cheng AL , et al. Cabozantinib in patients with advanced and progressing hepatocellular carcinoma. N Engl J Med. 2018;379(1):54‐63. 29972759 10.1056/NEJMoa1717002 PMC7523244

[42] Abou‐Alfa GK , Schwartz L , Ricci S , et al. Phase II study of sorafenib in patients with advanced hepatocellular carcinoma. J Clin Oncol. 2006;24(26):4293‐4300. 16908937 10.1200/JCO.2005.01.3441

[43] Abou‐Alfa GK , Amadori D , Santoro A , et al. Safety and efficacy of sorafenib in patients with hepatocellular carcinoma (HCC) and Child‐Pugh a versus B cirrhosis. Gastrointest Cancer Res. 2011;4(2):40‐44. 21673874 PMC3109886

[44] Demir T , Lee SS , Kaseb AO . Systemic therapy of liver cancer. Adv Cancer Res. 2021;149:257‐294. 33579425 10.1016/bs.acr.2020.12.001

[45] Pressiani T , Boni C , Rimassa L , et al. Sorafenib in patients with Child‐Pugh class a and B advanced hepatocellular carcinoma: a prospective feasibility analysis. Ann Oncol. 2013;24(2):406‐411. 23041587 10.1093/annonc/mds343

[46] Marrero JA , Kudo M , Venook AP , et al. Observational registry of sorafenib use in clinical practice across Child‐Pugh subgroups: the GIDEON study. J Hepatol. 2016;65(6):1140‐1147. 27469901 10.1016/j.jhep.2016.07.020

[47] Miller AA , Murry DJ , Owzar K , et al. Phase I and pharmacokinetic study of sorafenib in patients with hepatic or renal dysfunction: CALGB 60301. J Clin Oncol. 2009;27(11):1800‐1805. 19255312 10.1200/JCO.2008.20.0931 PMC2668705

[48] Vogel A , Frenette C , Sung M , et al. Baseline liver function and subsequent outcomes in the phase 3 REFLECT study of patients with unresectable hepatocellular carcinoma. Liver Cancer. 2021;10(5):510‐521. 34721512 10.1159/000516490 PMC8527908

[49] Ueshima K , Nishida N , Hagiwara S , et al. Impact of baseline ALBI grade on the outcomes of hepatocellular carcinoma patients treated with lenvatinib: a multicenter study. Cancers (Basel). 2019;11(7):952. 31284682 10.3390/cancers11070952 PMC6678474

[50] Dipasquale A , Marinello A , Santoro A . A comparison of lenvatinib versus sorafenib in the first‐line treatment of unresectable hepatocellular carcinoma: selection criteria to guide physician's choice in a new therapeutic scenario. J Hepatocell Carcinoma. 2021;8:241‐251. 33884259 10.2147/JHC.S270532 PMC8055282

[51] Yukimoto A , Hirooka M , Hiraoka A , et al. Using ALBI score at the start of sorafenib treatment to predict regorafenib treatment candidates in patients with hepatocellular carcinoma. Jpn J Clin Oncol. 2019;49(1):42‐47. 30380075 10.1093/jjco/hyy153

[52] Wang HW , Chuang PH , Su WP , et al. On‐treatment albumin‐bilirubin grade: predictor of response and outcome of sorafenib‐regorafenib sequential therapy in patients with unresectable hepatocellular carcinoma. Cancers (Basel). 2021;13(15):3758. 34359658 10.3390/cancers13153758 PMC8345148

[53] Kim HD , Bang Y , Lee MA , et al. Regorafenib in patients with advanced Child‐Pugh B hepatocellular carcinoma: a multicentre retrospective study. Liver Int. 2020;40(10):2544‐2552. 32563213 10.1111/liv.14573

[54] Yoo C , Byeon S , Bang Y , et al. Regorafenib in previously treated advanced hepatocellular carcinoma: impact of prior immunotherapy and adverse events. Liver Int. 2020;40(9):2263‐2271. 32449588 10.1111/liv.14496

[55] Demirtas CO , D'Alessio A , Rimassa L , Sharma R , Pinato DJ . ALBI grade: evidence for an improved model for liver functional estimation in patients with hepatocellular carcinoma. JHEP Rep. 2021;3(5):100347. 34505035 10.1016/j.jhepr.2021.100347 PMC8411239

[56] El‐Khoueiry AB , Meyer T , Cheng AL , et al. Safety and efficacy of cabozantinib for patients with advanced hepatocellular carcinoma who advanced to Child‐Pugh B liver function at study week 8: a retrospective analysis of the CELESTIAL randomised controlled trial. BMC Cancer. 2022;22(1):377. 35397508 10.1186/s12885-022-09453-z PMC8994237

[57] Bang YH , Lee CK , Yoo C , et al. Real‐world efficacy and safety of cabozantinib in Korean patients with advanced hepatocellular carcinoma: a multicenter retrospective analysis. Ther Adv Med Oncol. 2022;14:17588359221097934. 35602405 10.1177/17588359221097934 PMC9118905

[58] Zhang H , Zhang W , Jiang L , Chen Y . Recent advances in systemic therapy for hepatocellular carcinoma. Biomark Res. 2022;10(1):3. 35000616 10.1186/s40364-021-00350-4 PMC8744248

[59] US Food and Drug Administration . FDA approves atezolizumab plus bevacizumab for unresectable hepatocellular carcinoma. Accessed February 16, 2023. https://www.fda.gov/drugs/resources‐information‐approved‐drugs/fda‐approves‐atezolizumab‐plus‐bevacizumab‐unresectable‐hepatocellular‐carcinoma

[60] US Food and Drug Administration . FDA approves tremelimumab in combination with durvalumab for unresectable hepatocellular carcinoma. Accessed February 16, 2023. https://www.fda.gov/drugs/resources‐information‐approved‐drugs/fda‐approves‐tremelimumab‐combination‐durvalumab‐unresectable‐hepatocellular‐carcinoma

[61] Zhu AX , Park JO , Ryoo BY , et al. Ramucirumab versus placebo as second‐line treatment in patients with advanced hepatocellular carcinoma following first‐line therapy with sorafenib (REACH): a randomised, double‐blind, multicentre, phase 3 trial. Lancet Oncol. 2015;16(7):859‐870. 26095784 10.1016/S1470-2045(15)00050-9

[62] Zhu AX , Baron AD , Malfertheiner P , et al. Ramucirumab as second‐line treatment in patients with advanced hepatocellular carcinoma: analysis of REACH trial results by Child‐Pugh score. JAMA Oncol. 2017;3(2):235‐243. 27657674 10.1001/jamaoncol.2016.4115

[63] US Food and Drug Administration . FDA grants accelerated approval to nivolumab for HCC previously treated with sorafenib. Accessed February 15, 2023. https://www.fda.gov/drugs/resources‐information‐approved‐drugs/fda‐grants‐accelerated‐approval‐nivolumab‐hcc‐previously‐treated‐sorafenib

[64] US Food and Drug Administration . FDA grants accelerated approval to pembrolizumab for hepatocellular carcinoma. Accessed February 15, 2023. https://www.fda.gov/drugs/fda‐grants‐accelerated‐approval‐pembrolizumab‐hepatocellular‐carcinoma

[65] Kudo M , Matilla A , Santoro A , et al. CheckMate 040 cohort 5: a phase I/II study of nivolumab in patients with advanced hepatocellular carcinoma and Child‐Pugh B cirrhosis. J Hepatol. 2021;75(3):600‐609. 34051329 10.1016/j.jhep.2021.04.047

[66] Kudo M , Finn RS , Edeline J , et al. Updated efficacy and safety of KEYNOTE‐224: a phase II study of pembrolizumab in patients with advanced hepatocellular carcinoma previously treated with sorafenib. Eur J Cancer. 2022;167:1‐12. 35364421 10.1016/j.ejca.2022.02.009

[67] Finn RS , Ryoo BY , Merle P , et al. Pembrolizumab as second‐line therapy in patients with advanced hepatocellular carcinoma in KEYNOTE‐240: a randomized, double‐blind, phase III trial. J Clin Oncol. 2020;38(3):193‐202. 31790344 10.1200/JCO.19.01307

[68] Cheng AL , Qin S , Ikeda M , et al. Updated efficacy and safety data from IMbrave150: atezolizumab plus bevacizumab vs. sorafenib for unresectable hepatocellular carcinoma. J Hepatol. 2022;76(4):862‐873. 34902530 10.1016/j.jhep.2021.11.030

[69] Sangro B , Sarobe P , Hervas‐Stubbs S , Melero I . Advances in immunotherapy for hepatocellular carcinoma. Nat Rev Gastroenterol Hepatol. 2021;18(8):525‐543. 33850328 10.1038/s41575-021-00438-0 PMC8042636

[70] de Castro T , Jochheim LS , Bathon M , et al. Atezolizumab and bevacizumab in patients with advanced hepatocellular carcinoma with impaired liver function and prior systemic therapy: a real‐world experience. Ther Adv Med Oncol. 2022;14:17588359221080298. 35251317 10.1177/17588359221080298 PMC8891886

[71] D'Alessio A , Fulgenzi CAM , Nishida N , et al. Preliminary evidence of safety and tolerability of atezolizumab plus bevacizumab in patients with hepatocellular carcinoma and Child‐Pugh A and B cirrhosis: a real‐world study. Hepatology. 2022;76:1000‐1012. 35313048 10.1002/hep.32468 PMC9790703

[72] Rizzo A , Ricci AD , Di Federico A , et al. Predictive biomarkers for checkpoint inhibitor‐based immunotherapy in hepatocellular carcinoma: where do we stand? Front Oncol. 2021;11:803133. 34976841 10.3389/fonc.2021.803133 PMC8718608

[73] Rizzo A , Cusmai A , Gadaleta‐Caldarola G , Palmiotti G . Which role for predictors of response to immune checkpoint inhibitors in hepatocellular carcinoma? Expert Rev Gastroenterol Hepatol. 2022;16(4):333‐339. 35403533 10.1080/17474124.2022.2064273

[74] Viscardi G , Tralongo AC , Massari F , et al. Comparative assessment of early mortality risk upon immune checkpoint inhibitors alone or in combination with other agents across solid malignancies: a systematic review and meta‐analysis. Eur J Cancer. 2022;177:175‐185. 36368251 10.1016/j.ejca.2022.09.031

[75] Di Federico A , Rizzo A , Carloni R , et al. Atezolizumab‐bevacizumab plus Y‐90 TARE for the treatment of hepatocellular carcinoma: preclinical rationale and ongoing clinical trials. Expert Opin Investig Drugs. 2022;31(4):361‐369. 10.1080/13543784.2022.2009455 34798793

[76] Murray CJ , Atkinson C , Bhalla K , et al. The state of US health, 1990–2010: burden of diseases, injuries, and risk factors. JAMA. 2013;310(6):591‐608. 23842577 10.1001/jama.2013.13805 PMC5436627

[77] GBD 2017 Cirrhosis Collaborators . The global, regional, and national burden of cirrhosis by cause in 195 countries and territories, 1990–2017: a systematic analysis for the global burden of disease study 2017. Lancet Gastroenterol Hepatol. 2020;5(3):245‐266. 31981519 10.1016/S2468-1253(19)30349-8 PMC7026710

[78] Liao WC , Hou MC , Chang CJ , Lee FY , Lin HC , Lee SD . Potential precipitating factors of esophageal variceal bleeding: a case‐control study. Am J Gastroenterol. 2011;106(1):96‐103. 20823836 10.1038/ajg.2010.342

[79] Mumtaz K , Ahmed US , Abid S , Baig N , Hamid S , Jafri W . Precipitating factors and the outcome of hepatic encephalopathy in liver cirrhosis. J Coll Physicians Surg Pak. 2010;20(8):514‐518. 20688015

[80] Sundaram V , Shaikh OS . Hepatic encephalopathy: pathophysiology and emerging therapies. Med Clin North Am. 2009;93(4):819‐836. vii. 19577116 10.1016/j.mcna.2009.03.009

[81] Berzigotti A , Garcia‐Tsao G , Bosch J , et al. Obesity is an independent risk factor for clinical decompensation in patients with cirrhosis. Hepatology. 2011;54(2):555‐561. 21567436 10.1002/hep.24418 PMC3144991

[82] D'Amico G , De Franchis R , Cooperative Study Group . Upper digestive bleeding in cirrhosis. Post‐therapeutic outcome and prognostic indicators. Hepatology. 2003;38(3):599‐612. 12939586 10.1053/jhep.2003.50385

[83] Simbrunner B , Beer A , Woran K , et al. Portal hypertensive gastropathy is associated with iron deficiency anemia. Wien Klin Wochenschr. 2020;132(1–2):1‐11. 10.1007/s00508-019-01593-w PMC6978296 31912289

[84] Intagliata NM , Caldwell SH , Tripodi A . Diagnosis, development, and treatment of portal vein thrombosis in patients with and without cirrhosis. Gastroenterology. 2019;156(6):1582‐1599.e1. 30771355 10.1053/j.gastro.2019.01.265

[85] Aithal GP , Palaniyappan N , China L , et al. Guidelines on the management of ascites in cirrhosis. Gut. 2021;70(1):9‐29. 33067334 10.1136/gutjnl-2020-321790 PMC7788190

[86] Marciano S , Díaz JM , Dirchwolf M , Gadano A . Spontaneous bacterial peritonitis in patients with cirrhosis: incidence, outcomes, and treatment strategies. Hepat Med. 2019;11:13‐22. 30666172 10.2147/HMER.S164250 PMC6336019

[87] Francoz C , Durand F , Kahn JA , Genyk YS , Nadim MK . Hepatorenal syndrome. Clin J Am Soc Nephrol. 2019;14(5):774‐781. 30996046 10.2215/CJN.12451018 PMC6500947

[88] Soulaidopoulos S , Goulis I , Giannakoulas G , et al. Hepatopulmonary syndrome is associated with the presence of hepatocellular carcinoma in patients with decompensated cirrhosis. Ann Gastroenterol. 2017;30(2):225‐231. 28243044 10.20524/aog.2016.0117 PMC5320036

[89] Zardi EM , Abbate A , Zardi DM , et al. Cirrhotic cardiomyopathy. J Am Coll Cardiol. 2010;56(7):539‐549. 20688208 10.1016/j.jacc.2009.12.075

[90] Ferenci P . Hepatic encephalopathy. Gastroenterol Rep (Oxf). 2017;5(2):138‐147. 28533911 10.1093/gastro/gox013 PMC5421503

[91] Randall B . Fatty liver and sudden death. A review. Hum Pathol. 1980;11(2):147‐153. 6105125 10.1016/s0046-8177(80)80133-x

[92] Penttila A . Sudden and unexpected natural deaths of adult males. An analysis of 799 forensic autopsies in 1976. Forensic Sci Int. 1980;16(3):249‐259. 7203323 10.1016/0379-0738(80)90210-8

[93] Llovet JM , Villanueva A , Marrero JA , et al. Trial design and endpoints in hepatocellular carcinoma: AASLD consensus conference. Hepatology. 2021;73:158‐191. 32430997 10.1002/hep.31327

[94] Zhang H , Du X , Dong H , et al. Risk factors and predictive nomograms for early death of patients with advanced hepatocellular carcinoma: a large retrospective study based on the SEER database. BMC Gastroenterol. 2022;22(1):348. 35854221 10.1186/s12876-022-02424-5 PMC9297630

[95] Cabibbo G , Celsa C , Enea M , et al. Progression‐free survival early assessment is a robust surrogate endpoint of overall survival in immunotherapy trials of hepatocellular carcinoma. Cancers (Basel). 2020;13(1):90. 33396833 10.3390/cancers13010090 PMC7796103

[96] Gronkjaer LL , Lauridsen MM . Quality of life and unmet needs in patients with chronic liver disease: a mixed‐method systematic review. JHEP Rep. 2021;3(6):100370. 34805816 10.1016/j.jhepr.2021.100370 PMC8585663

[97] Korean Liver Cancer A , National Cancer C . 2018 Korean Liver Cancer Association‐National Cancer Center Korea practice guidelines for the management of hepatocellular carcinoma. Gut Liver. 2019;13(3):227‐299. 31060120 10.5009/gnl19024 PMC6529163

[98] Li L , Mo FK , Chan SL , et al. Prognostic values of EORTC QLQ‐C30 and QLQ‐HCC18 index‐scores in patients with hepatocellular carcinoma–clinical application of health‐related quality‐of‐life data. BMC Cancer. 2017;17(1):8. 28052758 10.1186/s12885-016-2995-5 PMC5209840

[99] Heffernan N , Cella D , Webster K , et al. Measuring health‐related quality of life in patients with hepatobiliary cancers: the functional assessment of cancer therapy‐hepatobiliary questionnaire. J Clin Oncol. 2002;20(9):2229‐2239. 11980994 10.1200/JCO.2002.07.093

[100] Gandhi S , Khubchandani S , Iyer R . Quality of life and hepatocellular carcinoma. J Gastrointest Oncol. 2014;5(4):296‐317. 25083303 10.3978/j.issn.2078-6891.2014.046 PMC4110497

[101] Muzellec L , Bourien H , Edeline J . Patients' experience of systemic treatment of hepatocellular carcinoma: a review of the impact on quality of life. Cancers (Basel). 2021;14(1):179. 35008343 10.3390/cancers14010179 PMC8749998

[102] Galle PR , Finn RS , Qin S , et al. Patient‐reported outcomes with atezolizumab plus bevacizumab versus sorafenib in patients with unresectable hepatocellular carcinoma (IMbrave150): an open‐label, randomised, phase 3 trial. Lancet Oncol. 2021;22(7):991‐1001. 34051880 10.1016/S1470-2045(21)00151-0

[103] Gkika E , Grosu AL , Macarulla Mercade T , et al. Tumor treating fields concomitant with sorafenib in advanced hepatocellular cancer: results of the hepanova phase II study. Cancers (Basel). 2022;14(6):1568. 35326718 10.3390/cancers14061568 PMC8946145

